# Expectations of patients for the implementation of new nursing technology

**DOI:** 10.1093/eurpub/ckac131.178

**Published:** 2022-10-25

**Authors:** R Klawunn, ML Dierks

**Affiliations:** Institute of Epidemiology, Social Medicine and Health System Research, Hannover Medical School, Hannover, Germany

## Abstract

**Background:**

New technologies, including robots, incidence detection or patient mobilization units, are increasingly assumed to support nurses in their routines while improving care quality. In the Nursing Care Centre Hanover study (funded by the German ministry of education and research), new technologies are implemented in a hospital ward and used by nurses in their routines. As part of this study, hospital patients were interviewed regarding the question: What are patients’ expectations for the implementation of new technology into care delivery?

**Methods:**

Between August 2019 and February 2020, 17 semi-structured interviews were conducted with patients from the project ward. To stimulate a response, 8 presentation of technologies by video and text (3 per interview) were given during the interviews. Interviews were recorded, transcribed and coded by evaluative, qualitative content analysis. The coded material was then interpreted in light of the research question.

**Results:**

Patients anticipate positive and negative effects of new technology concerning themselves, but they also expect effects on nurses: Health, safety and health service quality improvements might be positive effects for patients, but they are concerned about emerging threads to health by unintended consequences. They raise concerns about the possible inabilities of elder patients to use technology properly. Patients expect physical and emotional stress release for nurses when using technology, but they fear the replacement of nurses. This would have negative consequences for patients, like social isolation due to being cared for by machines.

**Conclusions:**

Patients have ambivalent perceptions of new technologies in nursing care. They have a differentiated view of possible consequences, not merely for themselves but also for nurses. In general, they are positive about the implementation, but this must be carried out under certain conditions, so that technology is used in a supportive, but not replacing, manner.

**Key messages:**

• The patients’ perspective must be take into account for the implementation of nursing technology to avoid negative, unintended consequences.

• Inclusion and consideration of older patients and their technology skills can be a relevant factor for advancing the adoption of new technology into care delivery.

